# High Agreement and High Prevalence: The Paradox of Cohen’s Kappa

**DOI:** 10.2174/1874434601711010211

**Published:** 2017-10-31

**Authors:** Slavica Zec, Nicola Soriani, Rosanna Comoretto, Ileana Baldi

**Affiliations:** 1 Department of Cardiac, Thoracic and Vascular Sciences, Unit of Biostatistics, Epidemiology and Public Health, University of Padova, Padova, Italy; 2 Department of Statistics and quantitative methods, University of Milan, Bicocca, Italy

**Keywords:** Agreement statistics, Cohen's Kappa, Gwet’s AC1, Concordance analysis, Inter-rater agreement, Quality assessment of RCT

## Abstract

**Background:**

Cohen's Kappa is the most used agreement statistic in literature. However, under certain conditions, it is affected by a paradox which returns biased estimates of the statistic itself.

**Objective:**

The aim of the study is to provide sufficient information which allows the reader to make an informed choice of the correct agreement measure, by underlining some optimal properties of Gwet’s AC1 in comparison to Cohen’s Kappa, using a real data example.

**Method:**

During the process of literature review, we have asked a panel of three evaluators to come up with a judgment on the quality of 57 randomized controlled trials assigning a score to each trial using the Jadad scale. The quality was evaluated according to the following dimensions: adopted design, randomization unit, type of primary endpoint. With respect to each of the above described features, the agreement between the three evaluators has been calculated using Cohen’s Kappa statistic and Gwet’s AC1 statistic and, finally, the values have been compared with the observed agreement.

**Results:**

The values of the Cohen’s Kappa statistic would lead to believe that the agreement levels for the variables Unit, Design and Primary Endpoints are totally unsatisfactory. The AC1 statistic, on the contrary, shows plausible values which are in line with the respective values of the observed concordance.

**Conclusion:**

We conclude that it would always be appropriate to adopt the AC1 statistic, thus bypassing any risk of incurring the paradox and drawing wrong conclusions about the results of agreement analysis.

## 1 INTRODUCTION

The analysis of intra- and inter-observer agreement is applied in many areas of clinical research [1-4]: from the diagnosis to evaluation of quality of experimental studies [5, 6]. As for the latter, the literature is unanimous in considering that low-quality trials, conducted using inadequate methodological approach, are often associated with the over-estimated treatment effects [5, 7]. These distortions can lead to errors at every level of decision making in health care, from individual treatment to definition of national public health policies. Quality assessments of trials are generally conducted by different parties (raters or evaluators) who are asked to verify, through appropriate checklists or scales [8-12], if the studies meet the predefined quality criteria. The agreement analysis, in these cases, does not only have the purpose to establish the reproducibility of the evaluations but, above all, to provide information about the role of the subjective component in definition of classifications and scores. It is important to note that the evaluation of the subjective component in rating is closely linked to sociometric and psychometric research field, from which the concordance measures originated in the first place [13-15].

The Cohen’s Kappa statistic [16] is the most used agreement measure in literature. This statistic does not have absolute applicability since it suffers from a particular paradox already known in literature [17-19]. Under special conditions [20, 21] and even in presence of a strong inter- or intra- rater agreement, the Kappa statistic tends to assume low values, often leading to conclude that no agreement is present. Consequently, the use of the Kappa statistics in presence of this paradox tends to affect the findings in terms of real reproducibility of measurement operations or lead to biased assessment results.

Among the alternative agreement measures to the Cohen’s Kappa [22-24], the statistic known as Agreement Coefficient 1 (AC1) given by Gwet [25] has proven to be most robust to this paradox [20, 21].

The purpose of this work is to provide sufficient information which allows the reader to make an informed choice of the correct agreement measure.

In the following sections Cohen’s kappa statistic will be introduced in its general formulation, with more than two categories and more than two evaluators, and conditions that lead to the paradox will be briefly described. The statistic AC1 will be subsequently introduced. Finally, a working sample, drafted from a reproducibility study among the evaluators of the quality of a clinical trial, will be used to show the behavior of the two statistics - both in presence and absence of the paradox.

### 1.1 The Cohen’s Kappa Statistic

In order to recall the concept and the construction of Cohen's Kappa statistic, let us suppose that we intend to compare the classifications of N subjects performed by R evaluators concerning K possible outcome categories (Table **1**). The generic R_ij_ indicates the number of evaluators that allocate the subject *i* to the category *j*.

The Kappa statistic, as well as other statistics of the same type [22-24], measure the concordance in data as a part of the agreement that cannot be observed due to mere chance and is defined [16] as:

(1) $$K\frac{P_{a}-P_{e|\;K}}{1-P_{e|\;K}}$$

in which:

(2) $$P_{a}\frac{1}{N}\sum_{i=1}^{N}\sum_{k=1}^{K}\frac{R_{\mathrm{ik}}(R_{\mathrm{ik}}-1)}{R(R-1)},\;$$

is the agreement observed in the data, while the expected agreement in case of random assignment is given by:

(3) $$P_{e|K}=\sum_{K=1}^{K}\sum_{r=2}^{R}{\left(-1\right)}^{r}\sum_{i_{1},i_{2},.......i_{r}}\prod_{j=1}^{r}{\mathrm{pki}}_{j}$$

The term *p_ki_j__*, for *j*=1,…, *r*, represents the portion of the subjects allocated to the category *k* by the evaluator *j*. The expression [3] is referring to the extension of Cohen’s Kappa to a more general case with more than two evaluators and more than two categories [26].

The statistics can assume any value from $-\frac{P_{e|K}}{1-P_{e|K}}$ and 1. Values greater than 0.6 are considered as indicators of high agreement, while values inferior to 0.4 or negative are indicators of discordance [27].

### 1.2 Cohen’s Kappa Paradox

The paradox undermines the assumption that the value of the Kappa statistic increases with the agreement in data. In fact, this assumption is weakened - sometimes even contradicted - in presence of strong differences in prevalence of possible outcomes [17]. These conclusions stem from sensitivity studies [20, 21], conducted for the case with two evaluators and two categories, who have analyzed the behavior of the Kappa statistic considering various interactions between the prevalence of outcomes in population, and the sensitivity and the specificity of evaluators (where sensitivity and specificity are defined as the probabilities that the evaluators correctly allocate a subject in one of the outcomes). Sensitivity studies have shown that the effects of the paradox arise in the presence of the outcomes with very high prevalence and/or considerable differences in classification probabilities. The paradox, in other words, is present when the examined subjects tend to be classified to one of the possible outcomes. This is either due to the nature the outcome itself and its high prevalence, or because at least one of the evaluators tends to assign more frequently to one specific outcome.

### 1.3 AC1 Statistic

The statistic AC1 has been proposed by Gwet [25] as an alternative agreement measure to Cohen’s Kappa statistic. According to Gwet [20], the reason why the Kappa statistic is exposed to the paradox lies in the inadequacy of the formula (3) for the expected agreement calculation.

Intuitively, the formulation of the statistic AC1 [25, 28] is rather similar to Cohen’s Kappa statistic:

(4) $$\gamma_{1}=\frac{P_{a}-P_{e|\gamma_{1}}}{1-P_{e|\gamma_{1}}},$$

in which the observed agreement *P_a_* is defined exactly as in the expression (2), while the expected agreement is defined as:

(5) $$P_{e|\gamma_{1}}=\frac{1}{K-1}\sum_{k=1}^{K}{\hat{\pi }}_{k}(1-{\hat{\pi }}_{k}),$$

where ${\hat{\pi }}_{k}=\frac{1}{N}\sum_{i=1}^{N}\frac{R_{\mathrm{ik}}}{R}$ . It is defined in a way that it cannot assume values higher than 0.5 [20], even if a part of the evaluators classifies in a completely random manner, without any consideration of the characteristics of the subjects.

The variance of the AC1 statistics, indispensable for the construction of confidence intervals, is calculated through the expression (3), following Gwet [28].

## 2 METHODS

### 2.1 Case Study: Reproducibility of the Evaluation of Clinical Trial Quality

During the process of literature review [29], we have asked a panel of three evaluators to come up with a judgment on the quality of 57 randomized controlled trials (RCTs), assigning a score to each trial using the Jadad scale [9]. This scale assigns a score from zero to five to a trial and evaluates presence and adequacy of the double-blind design, presence and adequacy of randomization and a possible loss of subjects during the study. An RCT is considered of good quality if it gets a score equal to or greater than 3. To explore some design aspects, the evaluators were asked to classify the trial depending on the type of randomization unit (individual or community), the type of design adopted (parallel, factor or crossover) and the type of the primary endpoint (binary, continuous, survival or other). The classifications of the three evaluators are shown in Table **2**, where the Jadad score was dichotomized, distinguishing between good (> 3), and poor (<3) quality trial.

## 3 RESULTS

The graphs shown in Fig. (**1**) describe the effect of the paradox on Cohen's Kappa statistic. The curves, shown in black in Fig. (**1**), are the values of the Kappa statistic as a function of prevalence, considering different scenarios for different levels of agreement and observed sensitivity and specificity of the evaluators. Following the sensitivity studies [20, 21], the curves of Fig. (**1**) assume that the two evaluators have the same values for sensitivity and specificity and that these values coincide. As we can see, in all scenarios considered (hence independent on the observed correlation values, sensitivity and specificity) the paradox begins to be evident for values of prevalence higher than 60%.

On the other hand, AC1 statistic (whose values are shown in red) appears more robust under the paradox conditions. The values of the AC1 statistics are in line with the observed correlation values, hence do not seem to be particularly affected by the prevalence level.

With respect to each of the above described features, the agreement between the three evaluators has been calculated. Table **3** shows the observed agreement (*P_a_*), the Cohen’s Kappa statistic (*γ_k_*), the statistic *AC1* (*γ*_1_), and their respective confidence intervals at 95%.

The values ​​of the Cohen’s Kappa statistic would lead to believe that the agreement levels for the variables Unit, Design and Primary Endpoints are totally unsatisfactory. However, a simple "glance" with the relative values ​​of the observed concordance is enough to highlight the presence of paradox. The most likely explanation for the onset of the paradox can be given by high values, shown in Table **2**, taken from the levels "Individual", "Parallel" and "Continuous" for variables Unit, Design and Primary Endpoint. These values ​​have led to high probability of classification and hence to paradox affected values of ​​Kappa statistic. The AC1 statistic, on the contrary, shows plausible values which are in line with the respective values ​​of the observed concordance.

For the Jadad variable, we can observe that in the absence of paradox, the Kappa statistic and AC1 have quite similar values which are both consistent with the observed concordance.

## 4 DISCUSSION

In this study, the intention was to briefly present and discuss a paradox that afflicts a concordance measure widely used in literature. As we have previously pointed out, the risk to encounter this paradox should be taken into account by the researcher who uses Cohen’s Kappa statistic in order to adequately tailor agreement analysis. Even in simple cases with only two evaluators and two outcomes, the paradox tends to occur if, at equal sensitivity and specificity of the evaluators, the prevalence of one of the results is above 60%, as seen in Fig. (**1**) graphs. Consequently, it is reasonable to assume that if we are dealing with a setting in which one of the outcomes has prevalence levels over 60%, then Kappa statistic might lead to biased conclusions and hence it is more suitable to use an alternative agreement statistic, such as AC1, less sensitive to this problem.

The AC1 statistic is not the only one that presents robustness properties to the paradox. The Alpha Aickin statistic [24] is another tool that has very similar properties to the AC1 [30]. In this study we have chosen to focus on the AC1 statistic since it is comparable with the Cohen's Kappa from the conceptual point of view [30] and computationally less intensive than of Alpha Aickin.

The use of AC1 statistics would also be advisable in all cases in which the evaluators are subject to a high probability of classification to one of the possible outcomes. In this case it is crucial to distinguish between the prevalence and the probability of classification. Prevalence is the probability (in many cases unknown) that an individual chosen at random from the population presents a specific level/category of an outcome. The probability of classification is a subjective propensity of the evaluators to assign to a particular outcome. This means that there exist different sources of paradox and that not always high prevalence follows high probability of classification and vice versa. This aspect can be observed in the example from the previous section, in which the high values ​​are both expression of high prevalence, as for the variable Unit where it is reasonable that the "Individual" level is predominant compared to the level "Community", but also result from the fact that for the variable Design, the evaluators did not have sufficient expertise to distinguish less common designs compared to that of "Parallel" type.

Even in the absence of the paradox, as in the example of Jadad score, the AC1 statistics provides absolutely consistent values ​​and overlapping with the Cohen’s Kappa, which confirms the results found in the literature [21, 28].

## CONCLUSION

On the basis of literature review and case study findings, we can conclude and suggest to the reader that it might always be appropriate to adopt the AC1 statistics, thus bypassing any risk of incurring the paradox and drawing wrong conclusions about the results of agreement analysis.

## Figures and Tables

**Fig. (1):**
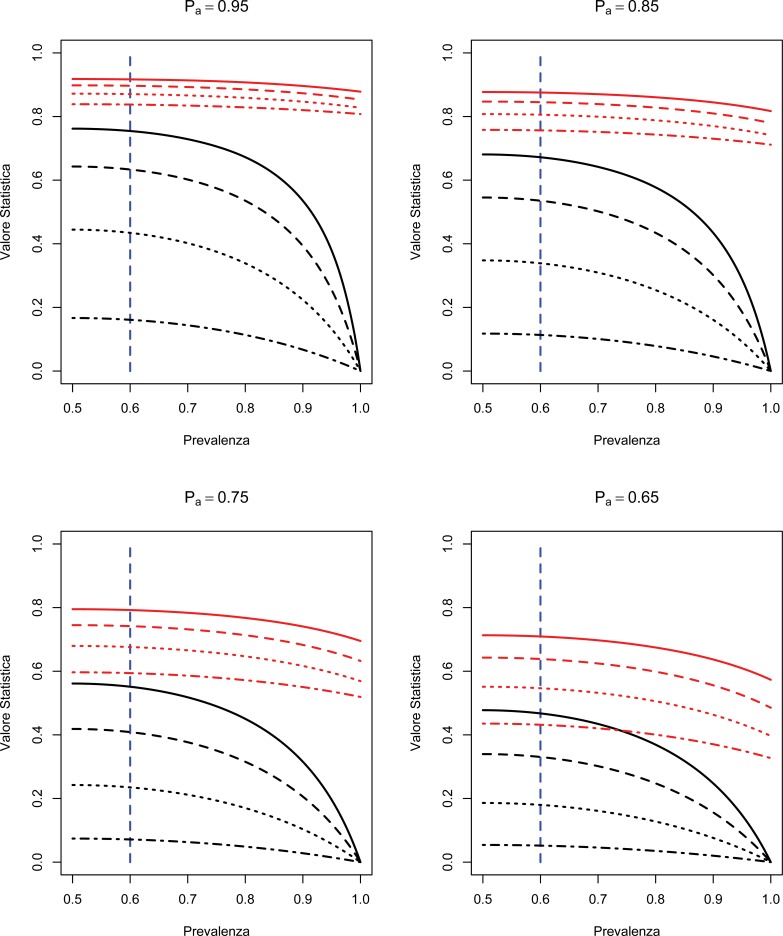
Cohen's Kappa (black lines) and AC1 (red lines) values computed by increasing the prevalence. The curves refer to several values of observed agreement (*P_a_*), and raters’ sensitivity and specificity. It is assumed that sensitivity and specificity values are equal and the same for both the raters.

**Table 1:** Distribution of N subjects for R raters and K outcomes.

Outcome						
Subject		1	2	…..	K	Total
	1	*R*_11_	*R*_12_	…..	*R*_1_*_K_*	*R*
	2	*R*_21_	*R*_22_	…..	*R*_2_*_K_*	*R*
				…..		* *
	*N*	*R*_N1_	*R*_N2_	…..	*R_NK_*	*R*
	Total	*R*_+1_	*R*_+2_	…..	*R_+K_*	N * R

**Table 2:** Results of the ratings carried out by the three raters on the characteristics investigated in the study.

**Variable**	**Evaluator 1**	**Evaluator 2**	**Evaluator 3**
**Unit**			
*Community*	4	0	6
*Individal*	53	57	51
**Design**			
*Crossover*	2	2	4
*Factorial*	9	3	8
*Parallel*	46	52	45
**Primary Endpoint**			
*Binary*	8	2	13
*Continuous*	42	31	43
*Survival*	3	7	1
*Other*	2	9	0
*Not specified*	2	8	0
**Jadad**			
*<3*	22	24	25
*≥3*	35	33	32

**Table 3:** Observed agreement (*P_a_*), Cohen's Kappa (*γ_k_*), AC1 (*γ*_1_) and their 95% confidence intervals computed on the ratings of the three raters.

	*P_a_*	*γ_k_*	*γ*_1_
**Randomization unit**	0.842 ( 0.747 -- 0.937 )	0.042 ( -1.000 -- 1.000 )	0.881 ( 0.725 -- 1.000 )
**Design**	0.719 ( 0.603 -- 0.836 )	0.230 ( -0.713-- 1.000 )	0.781 ( 0.682 -- 0.880 )
**Primary endpoint**	0.386 ( 0.260 -- 0.512 )	0.107 ( -0.203 -- 0.417 )	0.470 ( 0.439 -- 0.502 )
**Jadad**	0.871 ( 0.819 -- 0.924 )	0.735 ( 0.377 -- 1.000 )	0.750 ( 0.746 -- 0.754 )

## LIST OF ABBREVIATIONS

AC1: = Agreement Coefficient 1
RCT: = Randomized Control Trial
